# Supplementary material for: Content-rich biological network constructed by mining PubMed abstracts
Source: BMC Bioinformatics. 2004 Oct 8;5:147. doi: 10.1186/1471-2105-5-147 (PMC528731; doi:10.1186/1471-2105-5-147)
Supplement: Additional File 5 — The original Chilibot query results of the term "long-term potentiation (LTP)" and 22 other terms, limiting the latest references analyzed to the years 1990, 1995, 2000, and 2004. [file 1471-2105-5-147-S5.bz2 › chilibotAdditionalFile5/ltp2004/html/TRKB_PI-3K.html]

 


 **TRKB** and **PI-3K** 
  
Found 13 abstracts in PubMed,  **13 abstracts were retrieved and analyzed**.  


---

 Search Google  |
 PDF files only 
|  EDU domain only 

---

**Interactive relationship** (e.g. stimulation, inhibition, etc)

- Therefore, the PI 3 kinase  [ **PI-3K** ]  Akt pathway is co activated by NMDA and  **TrkB**  receptors.  Ref: 12373512 Amino Acids, 2002
- Using immunoprecipitation, PI3 kinase  [ **PI-3K** ]  and tyrosine phosphorylated PLC gamma1 and SHC were shown to be associated with tyrosine phosphorylated  **TrkB**  in response to both BDNF and NT 4.  Ref: 10486198 Exp Neurol, 1999

**Parallel relationship** (e.g. studied together, co-existance, homology, etc.)

- These observations suggest that  **TrkB**  signaling, via PI3 kinase  [ **PI-3K** ]  activation, plays an important role in controlling interneuron migration in the developing cerebral cortex.  Ref: 12070090 Development, 2002
- This indicates that the activation of the  **TrkB**  path through PI 3 kinase  [ **PI-3K** ]  is required for BDNF s survival promoting effects.  Ref: 12691830 Cancer Lett, 2003
- After binding to  **TrkB** , BDNF exerts this action by activating a PI 3 kinase  [ **PI-3K** ]  dependent pathway.  Ref: 10777780 J Neurosci, 2000
